# How I do it? Biportal endoscopic spinal surgery (BESS) for treatment of lumbar spinal stenosis

**DOI:** 10.1007/s00701-015-2670-7

**Published:** 2016-01-18

**Authors:** Chang Myong Choi, Je Tea Chung, Sang Jin Lee, Dae Jung Choi

**Affiliations:** Barun Spine Hospital, Yeoeudaebang-ro 1, Yeondeungpo-gu, Seoul Republic of Korea

**Keywords:** Biportal, Endoscopic spinal surgery, Spinal stenosis, Degenerative spine

## Abstract

**Background:**

Prevalent endoscopic spine surgeries have shown limitations especially in spinal stenosis (Ahn in Neurosurgery 75(2):124–133, 2014). Biportal endoscopic surgery is introduced to manage central and foraminal stenosis with its wide range of access angle and clear view.

**Methods:**

The authors provide an introduction of this technique followed by a description of the surgical anatomy with discussion on its indications and advantages. In particular, tricks to avoid complications are also presented.

**Conclusions:**

Effective circumferential and focal decompression were achieved in most cases without damage to the spinal structural integrity with preservation of muscular and ligamentous attachments. The biportal endoscopic spinal surgery (BESS) may be safely used as an alternative minimally invasive procedure for lumbar spinal stenosis (Figs. 1 and 2).

**Electronic supplementary material:**

The online version of this article (doi:10.1007/s00701-015-2670-7) contains supplementary material, which is available to authorized users.

## Relevant surgical anatomy

**Fig. 1:**
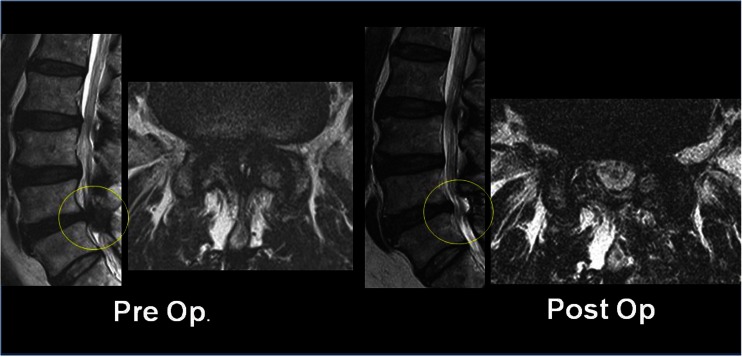
Pre- and postoperative findings of central stenosis

**Fig. 2:**
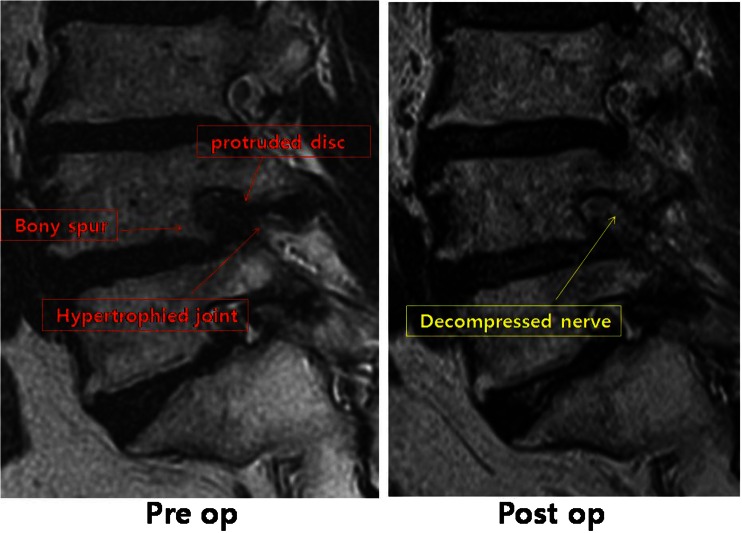
Pre- and postoperative findings of foraminal stenosis

**Fig. 3:**
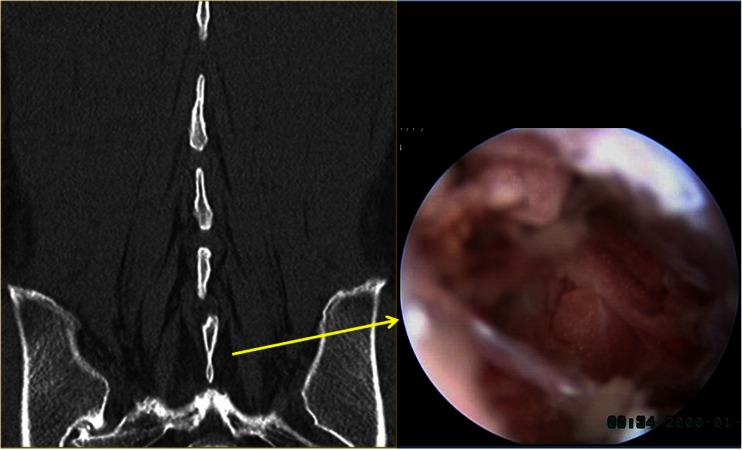
Multifidus muscle and spatium intermusculare with corresponding intraoperative endoscopic view

**Fig. 4:**
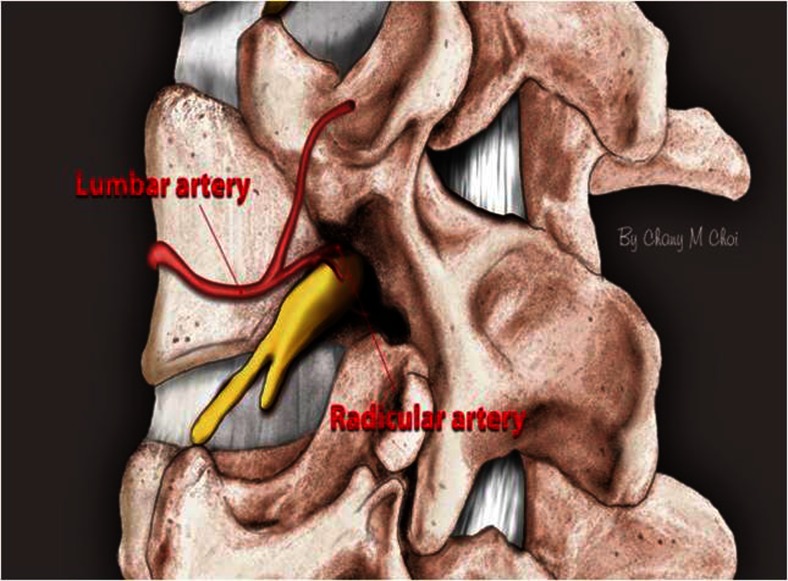
Schematic representation of the arteries around intervertebral foramen artist’s drawing: diagrammatic representation of the entry points for the portal sites and direction of the scope and instruments

Many minimally invasive procedures including various endoscopic procedures have been introduced to maintain the overall spinal structures (Figs. 1 and 2). The multifidus muscle is very important in its function as a stabilizer of spine and locomotor action. Even minimally invasive surgeries including various endoscopic procedures might damage the medial multifidus, which is innervated by the medial branch of the dorsal ramus with no segmental nerve supply as in the other paraspinal muscles [4]. This approach through spatium intermusculare with biportal endoscope and small cannula can prevent the erecta spinae from the injury by overdistracting procedures (Fig. 3). Furthermore, variable access angles permit wider and further view of the contralateral side. The paraspinal extraforaminal approach with this technique gives a wider view of the foraminal lesion avoiding injury of the exiting nerve and radicular artery (Fig. 4). With the proper biportal endoscopic surgical technique, the injuries to these structures can be avoided. By this procedure, we could treat all kinds of spinal stenosis including central, lateral recesses, and foraminal stenosis.

## Description of the technique

### Instruments

The standard arthroscopic facilities and conventional spine instruments such as Kerrison rongeurs, pituitary forceps, curettes, and high-speed diamond burrs are used.

### Room setup and patient positioning

The fluoroscopy unit and the video equipment for the endoscope. The procedure is performed under general or epidural anesthesia. The patient is placed in the prone position with the abdomen free over the radiolucent chest frame in a flexed position to open the interlaminar space and foramen.

### Endoscopic portals placement

**Fig. 5:**
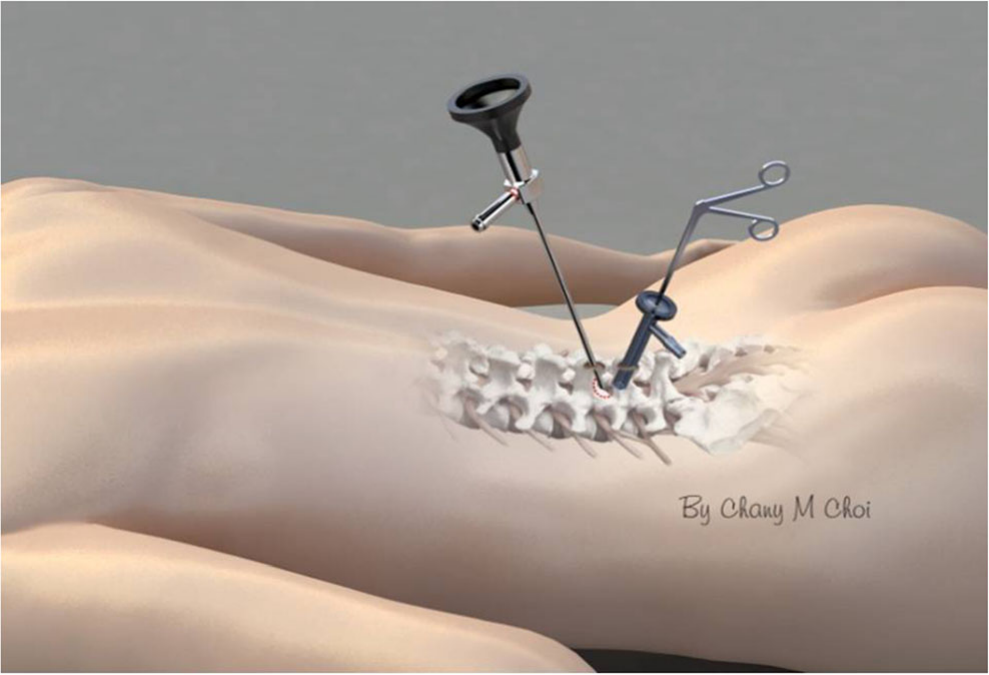
Biportal entry points for posterior approach

**Fig. 6:**
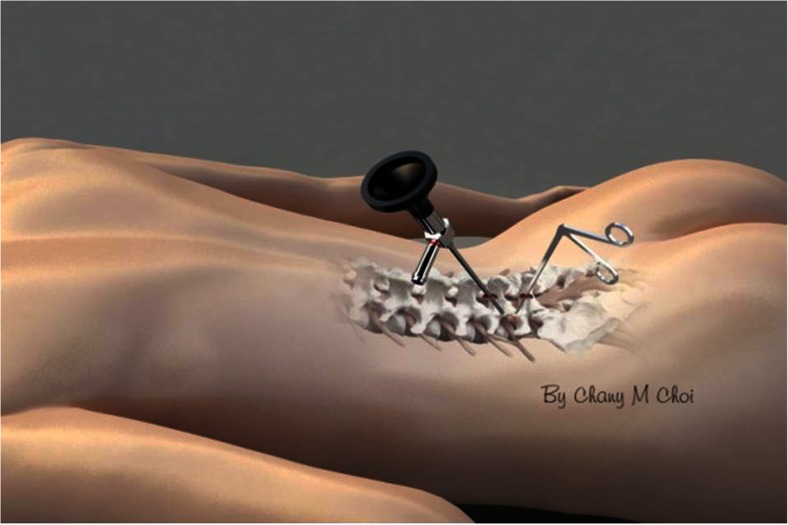
Biportal entry points for extraforaminal approach

Under image intensification, fluoroscopic confirmation of the level is made with a spinal needle inserted at the target area. Skin entry points are determined according to the lesion site and the patient’s anatomical variation. Two standard entry points are made at 1 cm above and below the disc space for a posterior approach (Fig. 5) and at the foramen level for the posterolateral approach (Fig. 6). The fascia is opened approximately 7 mm with a 15-blade scalpel along the skin crease followed by blunt muscle-splitting technique [2, 3] with a serial dilator touching the lamino-facet joint junction. Position is confirmed with biplanar fluoroscopy.

### Insertion of the endoscope and preparation of the surgical field

The posterior approach is accomplished via two portals through the intermuscular septum separating erector spinae and multifidus muscles using serial dilators. The multifidus muscle is saved by detaching the muscle from the lamina without injury with a blunt dissector to prepare a working space. This technique offers benefits over other techniques, such as the microendoscopic procedure, by creating a potential fatty space between the multifidus muscle in which to avoid crushing injury from over-retraction. We achieve a clear visual field with saline irrigation in this working space, which is used as the cavity-like joint space in arthroscopic surgery (Fig. 3).

### Laminotomy/medial facetectomy/ligamentum flavum removal

Thereafter, via a working cannula, conventional surgical instruments, such as the burr, punch, curette, and pituitary forceps, can be used freely in various access angles.

Depending on the pathology, ipsilateral decompression is performed first by performing hemilaminotomy with a drill and a Kerrison rongeur until the superior edge of the deep part of the ligamentum flavum in exposed. Hypertrophied facet joints and the lamina are undercut by drilling; then a blunt hook dissector is used to identify the plane between the ligament and the dura, ensuring that it is free from adhesions, and a curette and a punch can be used to peel off the ligaments and relieve the neural structures. If bilateral decompression is required, the midline of the spinal canal must first be confirmed by resecting the base of the spinous process with a high-speed drill. The scope can then be adjusted medially. Usually the base of the spinous process obstructs the placement of the scope, therefore it may need to be partially resected to secure sufficient working space. Once exposed, the ligamentum flavum can be detached from the contralateral lamina and then undercut with a burr. The entry to the contralateral side is performed dorsal to the dura with the ligamentum flavum intact for protection. Bony decompression is performed again using cranial and caudal laminotomy. Medal partial facetectomy of the contralateral superior articular process is performed to preserve the facet joint integrity. After bony decompression, thickened ligamentum flavum is resected with a curette to fully relieve the neural structures [5, 7]. The use of Kerrison rongeurs, a high-speed drill, and an ultrasonic bone cutter enables the lateral recess to be enlarged while keeping the facet joint intact. The endpoint of decompression is the outer edges of the bilateral nerve roots [6]. Continuous saline irrigation at 25 to 30 mmHg maintains a clear surgical view and preserves the epidural fat and vessels from damage, which may happen during the microendoscopic decompression surgery. This technique also avoids increased epidural hydrostatic pressure and subsequent increased intracranial pressure. Laminotomy and flavectomy is performed in a similar fashion as microscopic surgery, but bleeding is more effectively controlled by the radiofrequency bipolar system under continuous irrigation.

In case of foraminal stenosis, a working space around the foramen is achieved by meticulous dissection with a blunt dissector under clear vision and variable angle view. First, landing on the superior articular process is one of the important keys to the operation. The procedure begins at the safe extraforaminal area. Initial decompression of the superior articular process is performed sufficiently so that the exiting nerve is decompressed more safely without much manipulation. Out-in decompression around the nerve under wide view is of paramount importance [1].

## Indications

Moderate-to-severe spinal stenosis including central, lateral, and foraminal, moderate-to-large HNP, with or without mild instability; and grade I spondylolisthesis

## Limitations

1. For foraminal stenosis in severely collapsed disc space with bony spur, decompression without damaging the exiting nerve is difficult
2. Decompression alone is not adequate for unstable spine as it requires instrumentation for stabilization

## How to avoid complications

1. Use a controlled-pressure monitor irrigation pump to avoid over-increase of epidural hydrostatic pressure and subsequent increase of intracranial pressure: 25 to 30 mmHg for lumbar surgery. Keep continuous outflow of irrigating saline for clear view.
2. In order to prevent air embolism during the procedures, be aware of clearing air bubbles in the irrigation pump line.
3. In case of severe stenosis, there may be a dense adhesion of ligamentum flavum to the dura. In that case, frequent gentle tractions of ligamentum flavum from the dura with punch and pituitary forceps are helpful for spontaneous detachment. The careful insertion of a blunt hook over the dura will prevent tears in the dura, which leads to spontaneous adhesiolysis by saline irrigation into the epidural space between the dura and the overlying ligamentum flavum. If there is a dense adhesion between the dura and the ligamentum flavum, the outer layer is peeled off and the densely adhesed area is left over, keeping the dura intact.
4. The contralateral superior articular process and the upper lamina can be decompressed without nerve damage by undercutting the thick bony structure with burrs and the ultrasonic bone cutter. Then a small curette or a rongeur can be used to remove the remaining thin bony structures. These procedures are possible with clear vision under continuous saline irrigation.
5. In foraminal stenosis, the out-in approach starting at the extraforaminal area under wide and clear view is important.

### Preoperative considerations

Preoperative CT scan and a foraminal-view MRI can be studied to find out the feasibility of this technique in an individual patient.

## Specific intraoperative considerations

1. Saline irrigation pump is monitored to keep between 25 and 30 mmHg, depending on the patient’s condition to prevent increase of the epidural hydrostatic pressure and ICP with infusion of saline into the epidural space
2. Dural tears can be prevented by frequent piecemeal detachments of ligamentum flavum from the adhesed dura with saline irrigation into the potential space

## Postoperative considerations

Surgical drains are inserted and kept for 24 h after surgery until spontaneous bleeding is controlled. A muscle balance physiotherapy staged regimen is recommended on the second day following surgery.

## Specific perioperative considerations (pre and postop workup, instructions for the postop care)

Routinely check pre- and postoperative CT and MRI scans for thorough evaluation of the main pathology related to the patient’s symptoms. Patients should be counseled that there is likely to be some numbness or tingling over the dermatome of the nerve operated on, and neuroleptics may be prescribed until the symptoms subside.

## Key points

1. Lamina and foramen are distracted in prone, flexed position
2. Two entry points are determined under fluoroscope
3. Two approaches are possible, depending on the lesion site. The posterior approach is used for central and lateral recess stenosis. The posterolateral approach is chosen for foraminal stenosis.
  In case of foraminal stenosis, initially decompress the superior articular process and then decompress the herniated disc and bony spurs around the foramen. Be careful not to damage the exiting nerve.
4. Preserve multifidus muscles by going through the intermuscular septum without crushing or over-retraction injury.
5. Determine biportal entry points to get a wide-angle view with variable access angle according to the lesion.
6. Clear vision can be obtained under continuous saline irrigation.
  Hydrostatic pressure of an irrigation pump is monitored and kept between 25 and 30 mmHg to prevent an increase of epidural hydrostatic pressure and subsequent increase of ICP.
7. Free handling of surgical instruments such as burr and punch as doing open microsurgery
8. Easy learning curve for the surgeon who is acquainted with microscopic surgical anatomy
9. Broad indications: moderate-to-severe spinal stenosis with or without HNP, mild instability
10. Preserve epidural fat and vessels: Epidural fat can be preserved by the meticulous bleeding control with continuous saline irrigation and radiofrequency bipolar coagulation, without using suction tip over the epidural fat

## Electronic supplementary material

ESM 1 Video showing biportal endoscopic posterior approach for decompression of spinal stenosis. The operator is on the left side of the patient. (WMV 13360 kb)

## References

[1] Ahn Y (2014). Percutaneous endoscopic lumbar foraminotomy: an advanced surgical technique and clinical outcomes. Neurosurgery.

[2] Birkenmaier C, Komp M, Leu HF, Wegener B, Ruetten S (2013). The current state of disc surgery: review of controlled studies comparing full-endoscopic procedures for disc herniations to standard procedures. Pain Physician.

[3] Hesham MS (2013). Irrigation endoscopic discectomy: a novel percutaneous approach for lumbar disc prolapse. Eur Spine J.

[4] Hu ZJ, Fang XQ, Fan SW (2014). Iatrogenic injury to the erector spinae during posterior lumbar spine surgery: underlying anatomical considerations, preventable root causes, and surgical tips and tricks. Eur J Orthop Surg Traumatol.

[5] Komp M, Hahn P, Oezdemir S, Giannakopoulos A, Heikenfeld R, Kasch R, Merk H, Godolias G, Ruetten S (2015). Bilateral spinal decompression of lumbar central stenosis with the full-endoscopicInterlaminar versus microsurgical laminotomy technique: a prospective, randomized, controlled study. Pain Physician.

[6] Nomura K, Yoshida M (2012). Microendoscopic decompression surgery for lumbar spinal canal stenosis via the paramedian approach: preliminary results. Global Spine J.

[7] Wong AP, Smith ZA, Lall RR, Bresnahan LE, Fessler RG (2012) The microendoscopic decompression of lumbar stenosis: a review of current literature and clinical results. Hindawi Publishing Corporation Minim Invasive Surg. 2012, Article ID 325095: 11. doi:10.1155/2012/325095 10.1155/2012/325095 PMC3415081 22900163

